# Establishing a Cell-Based High-Content Screening Assay for TCM Compounds with Anti-Renal Fibrosis Effects

**DOI:** 10.1155/2018/7942614

**Published:** 2018-06-28

**Authors:** Xi-ting Wang, Xue-jiao Sun, Cheng Li, Yi Liu, Lan Zhang, Ya-dong Li, Qing-hua Wu, Shi-you Li, Yu Li

**Affiliations:** ^1^School of Traditional Chinese Medicine, Beijing University of Chinese Medicine, Beijing 100029, China; ^2^Key Laboratory of Genomic and Precision Medicine, Beijing Institute of Genomics, Chinese Academy of Sciences, No. 1 Beichen West Road, Chaoyang District, Beijing 100101, China

## Abstract

Renal fibrosis is thought to be the final common pathway leading to chronic kidney disease (CKD) and end-stage renal failure. Except for renal replacement therapy, no adequate treatment regimen is available; therefore studies on the treatment of renal fibrosis have attracted significant interest. In recent years, studies have shown that traditional Chinese medicine (TCM) may represent an attractive source to produce drugs with antifibrosis effects. The aim of this study was to establish a robust cell-based high-content screening (HCS) approach to identify TCM compounds with antifibrosis effects in NRK49F cells following TGF-*β*1 exposure. When designing the model, one of the most important steps involved the stability and reproducibility of this cell-based model. Therefore, we initially optimized the experimental parameters. Then, our HCS model was validated using SB525334, an inhibitor of the TGF-*β*1 receptor, and curcumin and emodin, two TCM compounds with well-documented anti-renal fibrosis activity. Subsequently, the proven reliable HCS model was used to screen a standard TCM compound library, which included 344 TCM molecules. Based on our HCS algorithm, a total of 16 compounds were identified to have prospective inhibitory activity. These compounds were further validated by verification experiments. Strikingly, eight compounds have been shown to inhibit renal fibrosis; six of them had rarely been described in the literature, namely, Ligustrazine, Glycyrrhizic acid, Astragaloside iv, Hydroxysafflor Yellow A, Crocin, and Gypenosides. To the best of our knowledge, this is the first study in which a HCS assay was performed to identify TCM compounds with anti-renal fibrosis effects. The HCS approach was successfully applied to screen active compounds and will be propitious to further anti-renal fibrosis drugs discovery research. Meanwhile, it may offer possibilities for identifying lead compounds for treating other diseases from registered Chinese herbal medicines.

## 1. Introduction

Fibrosis is defined as a wound-healing response that has gone out of control, resulting in substantial remodeling of the extracellular matrix (ECM) and formation of permanent scar tissue. Renal fibrosis is characterized by the accumulation of myofibroblasts and ECM components and is the principal process involved in the progression of chronic kidney disease (CKD) to end-stage renal disease (ESRD) [1]. Irrespective of the initial causes, renal fibrogenesis is a dynamic and converging process that consists of four overlapping phases: priming, activation, execution, and progression [2]. By activating the downstream Smad signaling pathway, TGF-*β*1 has long been considered a key mediator in the process of renal fibrosis [3].

The renin-angiotensin-aldosterone system blockade is central to the current treatment approach of patients with CKD for renoprotective effects aimed at preventing or slowing down the progression to ESRD [4]. However, there is an unmet need for novel therapeutics to combat the disease. Chinese herbal medicines have been considered invaluable resources in lead compound discovery. Moreover, recent studies have suggested that several Chinese herbal medicines have antifibrotic activity. For example, treatment with curcumin ameliorated renal fibrosis by reducing fibroblast proliferation and ECM accumulation mediated by PPAR-*γ* and Smad-dependent TGF-*β*1 signaling [5]. Epigallocatechin-3-gallate (EGCG) exerted protective effects against acute renal damage through its antioxidative effect by activating the Nrf2 signaling pathway [6]. In addition, Resveratrol inhibited renal interstitial fibrosis in diabetic nephropathy by regulating the AMPK/NOX4/ROS pathway [6].

Although traditional Chinese medicine (TCM) has shown a promising result in the treatment of renal fibrosis, in previous studies, large-scale drug screening approaches were limited, primarily due to their excessive costs and complex composition. Thus, establishing an efficient, rapid, and stable model for screening TCM compounds with anti-renal fibrotic effects was clearly warranted. In the past decade, automated microscopy techniques have become an important tool in drug discovery and development processes [7]. Indeed, automated microscopy-based assays have become an integral part of the drug discovery process because of the capacity to assess activity on a cellular level [7]. Successful disease-specific tailoring of therapeutics may be achieved by using an image-based screening approach, enabling analyzing a wide variety of cellular phenotypes. Such high-content screening (HCS) platforms employ fully automated microscopes and image analysis software, making it possible to quantify changes in cellular and subcellular properties, including cell area, morphology, actin fibers, and the intensity of focal adhesion [8]. In various studies, high-content whole-well imaging approaches have been described to evaluate drug activity [9–11]. However, in only a few reports, HCS was used for screening the activity of TCM compounds to treat renal fibrosis. Therefore, it is essential to use this method to increase coverage and screen a TCM compound library to identify novel TCM compounds with anti-renal fibrosis activity.

In this study, a cell-based model was established to screen TCM compounds with anti-renal fibrosis effect. As is known in many research works, *α*-smooth muscle actin (*α*-SMA) severs as well-characterized marker to identify pathologic fibroblasts [12]. Therefore, the expression of *α*-SMA was treated as king object of observation in this study to evaluate the drug activity. First, the optimum serum concentration and stimulation time of TGF-1 was determined. For the primary screening, the expression *α*-SMA was monitored in TGF-1-stimulated NRK49F cells. In this model, curcumin and emodin have been reported to have anti-renal fibrosis effects. Therefore, the HCS model was tested and validated by adding the TGF-*β*1 receptor blocker SB525334, curcumin, and emodin. Thus, we established a robust HCS model and screened a library that included 344 TCM compounds from the Beijing Institute of Genome Research (Beijing, China). A total of 16 compounds that showed prospective inhibition rates were selected. These compounds were further validated by verification studies. Since collagen III is the most abundant component in the ECM and is often used as a diagnostic marker for organ fibrosis, we evaluated the expression of collagen III in the verification experiments. Strikingly, eight compounds were identified to inhibit renal fibrosis; six of them had not been previously described in the literature, namely, Ligustrazine, Glycyrrhizic acid, Astragaloside iv, Hydroxysafflor Yellow A, Crocin, and Gypenosides. To the best of our knowledge, this is the first application of a HCS assay method used for this purpose. It is of utmost importance to screen for TCM compounds that can inhibit renal fibrosis for novel therapeutic purposes for CKD and ESRD patients. The established approach was successfully applied for screening of active TCM compounds and may offer a new means for identifying lead compounds from registered Chinese herbal medicines for the treatment of diseases.

## 2. Materials and Methods

### 2.1. Reagents and Materials

Recombinant human TGF-*β*1 was purchased from R&D systems (Shanghai, China). TGF-*β*1 receptor blocker SB525334 was purchased from Selleck Chemicals (Shanghai, China). The antibodies used included mouse anti-alpha smooth muscle actin antibody (Sigma Aldrich, St. Louis, MO, USA) and goat anti-mouse IgG (whole molecule)-FITC antibody, Thermo (Shanghai, China). BisBenzimide H33342 trihydrochloride (20mg/ml, Sigma Aldrich, St. Louis, MO, USA) was used for nuclear staining. Paraformaldehyde (PFA) was purchased from FuChen Chemical Reagent Factory (Tianjin, China). Bovine Serum Albumin (BSA) was from HWRK Chem Co., LTD (Beijing, China). Other materials included dimethyl sulfoxide (DMSO); DMEM, high glucose, and pyruvate (Invitrogen, State of California, USA); fetal bovine serum (FBS) (ExCell Bio, Shanghai, China); trypsin from bovine pancreas, EDTA, and Triton X-100 (Sigma Aldrich, St. Louis, MO, USA).

### 2.2. Cell Culture and HCS Assays

NRK49F cell lines were provided by the cellular center of basic medicine, Institute of Basic Medicine, Chinese Academy of Medical Sciences (Beijing, China), and were routinely maintained in DMEM, containing 5% FBS, 100U/mL penicillin, and 100 g/mL streptomycin at 37°C in a humidified atmosphere of 5% CO_2_. When the cell density reached 80%, cells were digested and passaged using 0.25% trypsin.

NRK49F cells were seeded in 96-well clear-bottom black plates (Corning, NY 14831, USA). Cells were seeded at a density of 6000 cells in 200 *μ*L medium per well and grown for 24h. Then, culture medium was removed, 200 *μ*L of fresh medium without serum was added to each well, and cells were incubated for another 24 hours. The cell number used ensured that the cells survived the repeated wash cycles in the subsequent experiment. Cells were divided into four groups: (a) model group: TGF-*β*1 solution (10 ng/mL) plus DMSO (0.1% final concentration); (b) TCM treatment group: TGF-*β*1 solution (10 ng/mL) and TCM compounds; (c) positive control: TGF-*β*1 solution (10 ng/mL) and SB525334 solution; (d) blank control: 5% FBS plus DMSO (0.1% final concentration).

Cells were stimulated with 10 ng/ml of recombinant TGF-*β*1 in DMEM with 5% FBS for 72 hours. Simultaneously, compounds were added to each well. The TCM compounds were added to the 96-well plates, and plates were incubated in a CO_2_ incubator and cultured for 3 days. Cells were fixed in prewarmed 4% PFA for 20 min at room temperature. Cells were rinsed 3 times with PBS at 200 *μ*l/well. Cells were permeabilized with 0.1% Triton X-100 in PBS (PBST) for 30 min at room temperature and blocked with 5% BSA in PBST for 1 hour. After blocking overnight at 4°C with 5% BSA in PBST, cells were incubated with *α*-SMA antibody (1:500). After washing 3 times with PBS, cells were incubated for 1.5 hours with fluorescein-conjugated affinity-purified goat anti-mouse IgG (1:500) and goat anti-rabbit IgG (1:500) at room temperature, protected from light. Then, cells were stained with Hoechst 33342 (20 *μ*g/ml) for 15 min and washed 3 times with PBS before image acquisition. PBS (100 *μ*L per well) was added to each well of a 96-well plate, and the plate was wrapped in tinfoil to prevent formation of air bubbles. Finally, the stained cells were subjected to high-content imaging analysis using a MetaXpress Micro XL imager (Sunnyvale, CA, USA).

### 2.3. Preparation of Compounds

Cuvettes containing 5 mg of SB525334 powder were centrifuged, sprayed with 75% alcohol, and transferred into a clean table for the preparation of the solution. DMSO was added to make a 10 mmol/l solution. After addition of DMSO, cuvettes were centrifuged. Then, 100 *μ*L of solution was extracted and added to 900 *μ*L of DMSO. Finally, the 1 mmol/L SB525334 solution was divided into 200 *μ*L/ tube and stored at -80°C for future experiments.

TCM compounds were provided by the Beijing Institute of Genomics in the Chinese Academy of Science (Beijing, China), dissolved in DMSO at 10 mM (for primary screening) or 36 mM (for secondary screening), and stored at -20°C.

### 2.4. Screening and Validation of Active Compounds with Antifibrosis Effects

For the primary screening, a layout-chart was made to determine the location of the different solutions within the 96-well plates. Cells were divided into four groups: model group, TCM treatment group, positive control group, and blank control group. To avoid the edge effect of the plate, a similar volume of culture medium was added to the surrounding wells of the 96-well plate. To identify compounds that inhibited TGF-*β*1-induced expression of *α*-SMA, the inhibition ratio (IR) was introduced as a measure of antifibrotic activity. The equation of inhibition ratio was as follows: *IR* = (compound − control)/(model − control), where compound, control, and model, respectively, represented the fluorescence intensity of TCM treatment group, blank control group, and model group.

For the verification experiment, another layout-chart was created to determine the location of the different solutions in 96-well plates. Expression levels of *α*-SMA and collagen III were monitored in TGF-1-stimulated NRK49F cells. The method of grouping was similar to the primary screening. To the well of TCM controls corresponding compound solution was added, and DMEM containing TGF-*β*1 and 10% FBS was used for making dilutions. The concentration of TCM compound controls was set between 0.89 *μ*M and 72 *μ*M.

### 2.5. Image Acquisition and Analysis

The nuclei of NRK49F cells were labeled with Hoechst 33342, and images of stained cells were acquired by high-content imaging analysis using a Molecular Device Image Xpress (Sunnyvale, CA, USA). The expression of *α*-SMA or collagen III was determined and quantified by changes in fluorescence intensity. Cell counts were assessed by nuclear imaging analysis, and the corresponding nuclei were localized by adjusting the parameters of the fluorescent image channel in the analysis software. The amplification of a certain region based on the nucleus was considered the area that was occupied by the cells. Expression of the corresponding protein of single cells was obtained by analyzing the fluorescence intensity of labeled proteins in that region.

### 2.6. Z' Factor

In the high-throughput screening assay, the feasibility of several experimental conditions and measurements was verified [13]. The Z' factor reflected both the dynamic range of the signal and data variation of the assay that was associated with signal measurements, and it was suitable for the assessment of assay quality [14]. The equation for calculating the Z' factor was as follows: Z' factor = 1 - (3 *∗* (*σ*p + *σ*n) / |(*μ*p- *μ*n)| ), where *σ* represented the sample variance, *μ* represented the average value, p represented the positive control, and n represented the negative control. In our study, the model group was adopted as the positive control, whereas the blank control was used as a negative control. A Z' factor with a value between 0.5 and 1 reflected high test results. In our study, the Z' factor was 0.55, which indicated that our method was suitable for a high-throughput assay.

### 2.7. Statistical Analysis

GraphPad Prism (GraphPad Software Inc., La Jolla, CA, USA) was used for all statistical analyses. P < 0.05 was considered statistically significant. Differences between groups were tested by one-way ANOVA with Dunnett's test. Data were expressed as the mean ±standard deviation.

## 3. Results and Discussion

### 3.1. Assay Outline and Parameter Optimization

**Figure 1:**
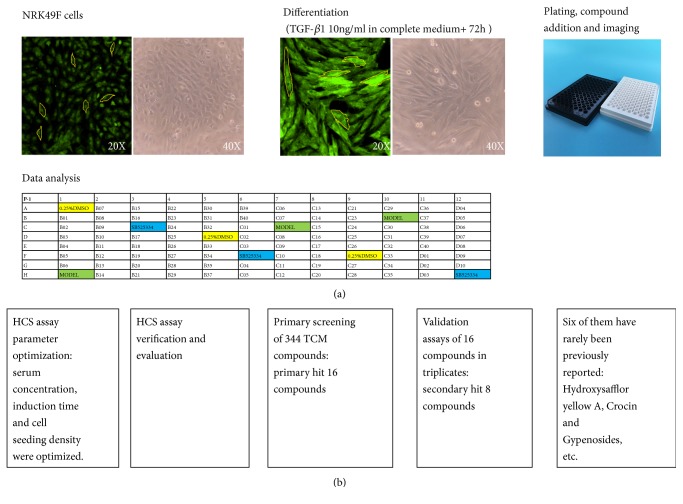
**Outline and workflow of the high-content screening assay. **(a) Schematic representation of NRK49F cells towards fibrosis and high-content screening (HCS) assay outline. NRK49F cells were seeded in 96-well plates at a density of 6,000 cells per well. Compounds were added, and after 72 h of culture, cells were fixed, stained, and subjected to high-content imaging analysis using MetaXpress Micro XL. A schematic approach for systematic identification of compounds with fibrosis inhibiting potential. Identification of the hit compounds was based on the above images using high-content analysis. The final selected compound from the screen included Hydroxysafflor Yellow A, Crocin, and Gypenosides. (b) Workflow of the study.

To facilitate HCS of novel compounds with anti-renal fibrosis activity, we analyzed the expression *α*-SMA and collagen III in TGF-*β*1-treated NRK49F cells by immunofluorescence. The workflow of the assay is presented in Figure 1. When running high-throughput assays, maintenance of consistency is of utmost importance to the accuracy of the results. In cell-based assays, achieving reproducibility is often more challenging compared to biochemical assays [15]. The first steps in establishing the methods for this assay were to select an NRK49F cell line and a fluorescent protein-expressing strain of *α*-SMA tuberculosis that would reliably function in a high-throughput live-cell-based assay. In the primary screening, an activated form of interstitial fibroblasts, *α*-smooth muscle actin-positive myofibroblasts, has been widely recognized as the major type of matrix-producing cells in the fibrotic kidney [16].

**Figure 2:**
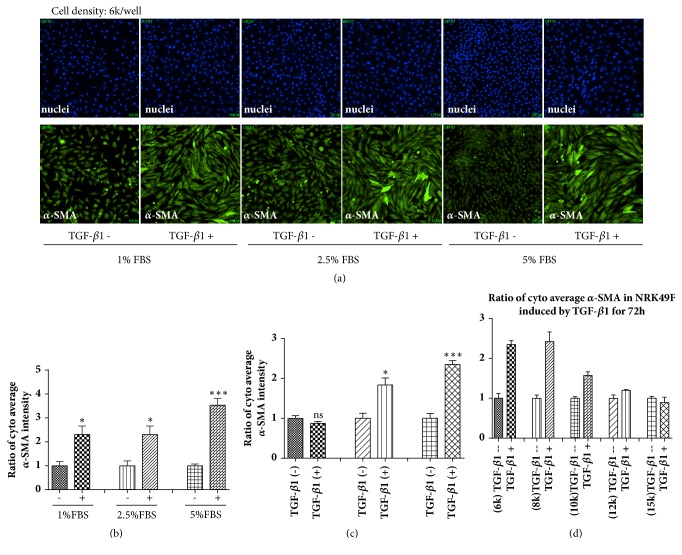
Optimization of high-content screening assay parameters. (a) Effect of serum on the expression of *α*-SMA in NRK49F cells with or without stimulation with TGF-*β*1 for 72 h by immunofluorescence. (b) Comparison of *α*-SMA expression in NRK49F cells with or without TGF-*β*1 stimulation for 72 h when treated with different serum concentrations. Comparison of *α*-SMA expression in NRK49F cells with or without TGF-*β*1 stimulation at different incubation times (c) and different concentrations (d). Means ± SD, ns: P > 0.05, *∗*: P < 0.05, *∗∗*: P < 0.01, *∗∗∗*: P < 0.001.

Since serum is rich in complex cytokines, it may interfere with the cellular state during the induction of the model. Therefore, optimization of the serum concentration was implemented (Figure 2(a)). The results showed that there was no significant difference in the expression of *α*-SMA between the model group and the blank control group when being serum-free. However, there was a significant difference between groups when serum was added, indicating that the transformation of NRK49F cells into fibroblasts and concomitant high expression of *α*-SMA required was serum dependent. Interestingly, with increasing serum concentrations, the expression of *α*-SMA significantly increased, indicating a dose-dependent effect of FBS (Figure 2(b)).

Subsequently, a set of experiments was performed to evaluate the time required to induce fibrotic changes. The induction time was set to 24, 48, and 72 h. The results indicated that there were significant differences in the expression of *α*-SMA between the blank control group and the model group at an induction time of 72 h (Figure 2(c)).

TGF-*β*1 induces the proliferation of fibroblasts; therefore, the role of TGF-*β*1 may be masked by cell contact inhibition if the seeding density is too high. Moreover, an excessive seeding density is not conducive to the HCS system for capturing and processing cell-based information. When the cell density was 6000 per well, a significant difference was observed in the expression of *α*-SMA between the blank control group and the model group (Figure 2(d)). Therefore, the cell density was set at 6000 cells per well.

A reliable HCS assay for screening active compounds with anti-renal fibrosis effects was established by optimizing parameters, including TGF-*β*1 concentration (10 ng/mL), exposure time (72 h), and cell density (6000 cells per well) in medium containing 5% FBS.

### 3.2. HCS Assay Model Verification and Evaluation

**Figure 3:**
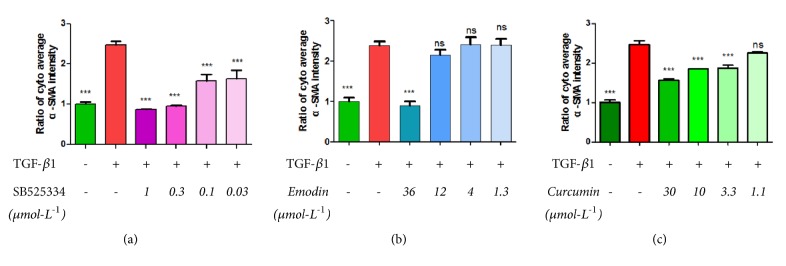
Expression of *α*-SMA in TGF-*β*1-stimulated NRK49F cells when treated with TGF-*β*1 receptor blocker SB525334 (a), emodin (b), and curcumin (c). Data are presented as the mean ± SD, ns: P > 0.05, *∗*: P < 0.05, *∗∗*: P < 0.01, *∗∗∗*: P < 0.001.

Once a reliable method of obtaining quantitative data from images had been established, the assay was tested for inter- and intra-experiment variability. TGF-*β*1 receptor blocker SB525334, curcumin, and emodin were used to test and validate the HCS model. Curcumin and emodin have previously been reported to have anti-renal fibrosis effects. The results are shown in Figure 3.

The results indicated that TGF-*β*1 receptor blocker SB525334, curcumin, and emodin to a certain extent inhibited *α*-SMA expression when compared with the model group, the curcumin group (P < 0.05), the SB525334 group, and the emodin group (P < 0.001). Moreover, three compounds, TGF-*β*1 receptor blocker SB525334, curcumin, and emodin, inhibited the proliferation of NRK49F cells (P < 0.001). Interestingly, the inhibition of SB525334 to *α*-SMA decreased with decreasing concentrations; however, curcumin and emodin did not show significant dose dependency.

Our results were in line with published data and verified that SB525334, curcumin, and emodin inhibited the expression of *α*-SMA in TGF-*β*-treated NRK-49F cells, thereby preventing NRK49F cells to transform into myofibroblasts. The Z factor of the HCS assay was 0.55, indicating that our method was suitable for a high-throughput assay. The results of our study suggested that a robust and reproducible assay was established, which will be highly useful for the discovery of TCM-derived compounds with anti-renal fibrosis activity.

### 3.3. HCS Screening and Verification

**Figure 4:**
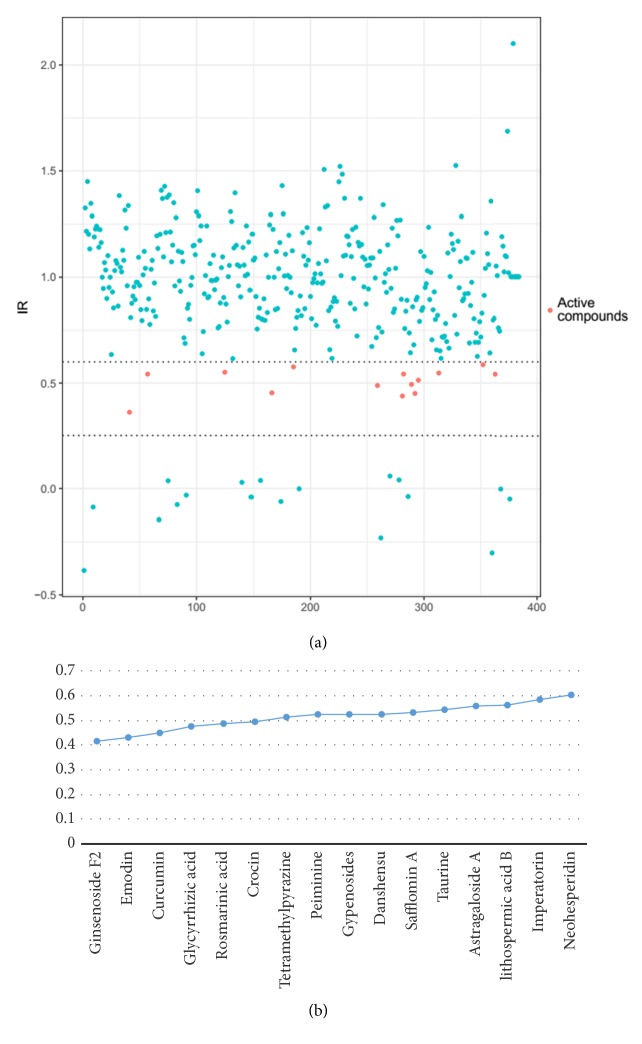
Primary screening of 344 compounds and hit compounds list with their inhibition ratio (IR). (a) Scatter plot of the primary screening compounds: red dots represent active compounds, whereas green dots represent negative compounds. (b) Figure showing the primary hit compounds and their IR.

A TCM library of 334 compounds was screened by HCS assay and their IR was calculated (Figure 4(a)). Compounds with an IR of <60% did not show obvious cytotoxic effects and were selected for further verification studies. In total, 16 primary hit TCM compounds were obtained (Figure 4(b)).

Validation assays were applied to verify the antifibrosis effects of TCM compounds. Since collagen III is the most abundant component in the ECM and is often used as a diagnostic marker for organ fibrosis, collagen III was added to evaluate the compounds in the validation assays. Different concentrations of the above-mentioned compounds were tested to evaluate their anti-renal fibrosis effects. Compounds were divided into five groups based on concentration: 72 *μ*mol·L^−1^, 24 *μ*mol·L^−1^, 8 *μ*mol·L^−1^, 2.67 *μ*mol·L^−1^, and 0.89 *μ*mol·L^−1^.

**Figure 5:**
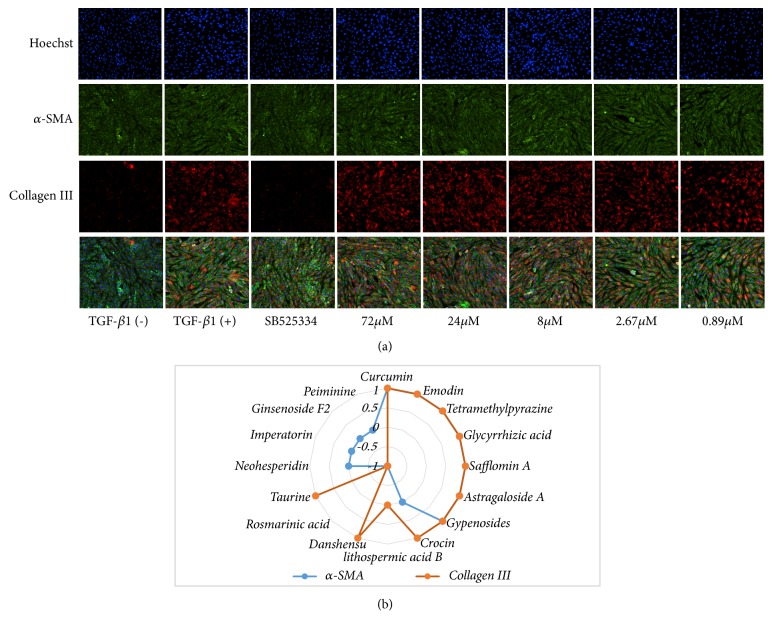
(a) Representative compound, Hydroxysafflor Yellow A, images of high-content screening analysis for the validation of compounds with antifibrosis effects. The effect of compounds on the expression of *α*-SMA (green) and collagen III (red) of TGF-*β*1-treated NRK49F cells was obtained. Nuclei were identified by Hoechst 33342 staining (blue). (b). Effect of TCM compounds on the expression of *α*-SMA and collagen III in TGF-*β*1-treated NRK49F cells. (“1” indicates obvious inhibitory effects; “-1” indicates no obvious inhibitory effects; “0” indicates that although there is no statistical difference, there is a decreasing trend.)

**Figure 6:**
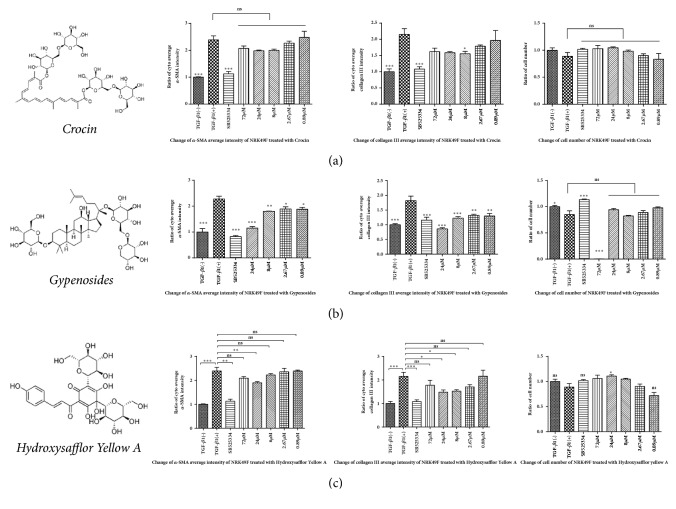
The structures and results of identified active compounds, Crocin, Gypenosides, and Hydroxysafflor Yellow A. The changes of *α*-SMA, collagen III average intensity, and cell number of NRK49F cells treated with (a) Crocin, (b) Gypenosides, and (c) Hydroxysafflor Yellow A.

Figure 5(a) shows images of the HCS analysis for the validation of compounds with antifibrosis effects for compound Hydroxysafflor Yellow A. A summary of the results of the validation assays is presented in Figure 5(b). The experimental results showed that Rosmarinic acid had no significant effects on the expression of *α*-SMA and collagen III. However, Peiminine, imperatorin, ginsenoside F2, and neohesperidin downregulated the expression of *α*-SMA (although there was no statistical difference compared with model group, there was a decreasing trend), but had no significant effects on the expression of collagen III. Salvianolic acid B, Danshensu, and taurine downregulated the expression of collagen III (taurine, P < 0.05, Salvianolic acid B, and Danshensu showed a decreasing trend, but no statistical differences were observed with the model group), but failed to inhibit the expression of *α*-SMA. In addition, curcumin, emodin, Ligustrazine, Glycyrrhizic acid, Hydroxysafflor Yellow A, Astragaloside, Gypenosides, and Crocin showed inhibitory effects on the expression of both *α*-SMA and collagen III. To our knowledge, Crocin, Gypenosides, and Hydroxysafflor Yellow A have not previously been reported. The data are presented in Figure 6.

## 4. Conclusions

Renal fibrosis, which includes glomerulosclerosis and tubulointerstitial fibrosis, involves the final manifestation of CKD. The pathogenesis of renal fibrosis is a complex process, which is characterized by the accumulation and deposition of ECM components. Targeting components of fibrogenic pathways has been under intense investigation as therapeutic approaches to inhibit or slow down the progressive loss of kidney function. Despite the extensive demand, current therapies for renal fibrosis treatment are limited [17]. In recent years, an increasing number of studies have suggested that TCM compounds represented promising sources to produce drugs with antifibrosis effects [17]. Previous research methods on the design of TCM-based drugs with anti-renal fibrosis effects mainly included animal models and* in vitro* models, which are often limited to testing a few compounds or only a single one. HCS enables measuring complex phenotypic outcomes that are more closely linked to disease states while also providing preliminary, cell-level assessment of certain aspects of ADMET during a primary screen [18]. Here, we established a robust cell-based HCS model in TGF-*β*1-induced NRK49F cells to screen active compounds with antifibrosis effects.

According to the related research on TGF-*β*, due to its central role in severe fibrotic diseases, TGF-*β* nevertheless remains an attractive therapeutic target, if targeted locally and during the fibrotic phase of disease [3, 12, 19–21]. Therefore, though the effects of TGF-*β* in renal fibrosis have not been confirmed in human with kidney disease, it can be appropriate to take TGF-*β* as inducer of cell-based model in the domain of compounds primary screening with anti-renal fibrosis effects. In our study, we used TGF-*β*1 to activate cells. Next, we tested the expression of *α*-SMA to assess the activation that was achieved after testing different serum concentrations, cell seeding densities, and treatment time. We found that *α*-SMA expression was significant when using 10 ng/mL TGF-*β*1, culture medium containing 5% FBS, cell density of 6000 cells per well, and a treatment time of 72 h. Then, the reported compounds with potential antifibrosis effects, curcumin, and emodin, along with TGF-*β*1 receptor blocker SB525334, were used to verify the model. The results suggested that we established a robust and stable cell-based HCS model for screening compounds with anti-renal fibrosis effects.

We successfully screened a TCM compound library and acquired live-cell images. In the primary screening, a total of 16 TCM compounds were found to inhibit the activation of fibroblasts. Next, we screened the 16 compounds with different concentration gradients, and in addition to *α*-SMA, the effects of collagen III were evaluated. Finally, eight compounds were found to inhibit the expression of *α*-SMA and collagen III. Among these compounds were curcumin and emodin, which have been described in previous studies [5, 22]. Here, we described six potent renal fibrosis inhibitors, including Ligustrazine, Glycyrrhizic acid, Astragaloside iv, Hydroxysafflor Yellow A, Crocin, and Gypenosides, that can block the differentiation of NRK49F cells* in vitro *towards fibrosis.

These TCM compounds were identified by the HCS model with primary and verification screening, which indicates the reliable outcome of our study. The results show that several identified compounds were less potent than TGF-*β*1 receptor blocker SB525334. However, in consideration of the strongly bifunctional role of TGF-*β* (profibrotic, but anti-inflammatory), it requires great care for the application of TGF-*β*-directed treatments [5, 22]. Thus, high inhibitory rate of TGF-*β* and high anti-renal fibrosis effects are primary but not unanimous corresponding. Besides, most Chinese medicine-derived compounds affect more than one target and do not correspond to the one-compound/one-target drug discovery paradigm [23]. As renal fibrosis is an enormously complex, dynamic process [2] and TCM proved to cure disease with multipathway mechanism, all these TCM have other potential renal protective effects than inhibition of TGF-*β* activity such as anti-inflammatory and antioxidative stress pathway [22, 24]. Therefore, it will be more precise and comprehensive to identify these TCM with anti-renal fibrosis effects through multiple aspects in the future. Overall, these compounds may serve as potential candidates for further research.

In summary, we established an* in vitro* model that was successfully applied to screen active compounds and may offer a new means for identifying lead compounds for treating renal fibrosis from registered Chinese herbal medicines. Of the screened compounds, six compounds, such as Gypenosides, may be promising for studying novel renal fibrosis drugs. Whether these compounds produce similar effects* in vivo* remains to be elucidated. Though, there are several limitations with our study, this study highlights that cell-based high-content assay method manifests good performance in TCM activity compounds screening and well application prospect in the domain of drug discovery.

## Data Availability

The underlying data related to this study is available upon request.

## Additional Points

*Limitations*. There are limitations with our study. As the active compounds were screened based on an* in vitro* model, the effects of these compounds* in vivo* in renal fibrosis have not been determined. Therefore, the further* in vivo *assay is worthwhile for the effects confirmation of these compounds.
